# Supplementary material for: High temperature delays and low temperature accelerates evolution of a new protein phenotype
Source: Nat Commun. 2024 Mar 29;15:2495. doi: 10.1038/s41467-024-46332-6 (PMC10980763; doi:10.1038/s41467-024-46332-6)
Supplement: Supplementary file 1 — Supplementary Info [file 41467_2024_46332_MOESM1_ESM.pdf]

Supplementary Information

High temperature delays and low temperature accelerates evolution of a new protein phenotype

Jia Zheng[1,2,3]*†, Ning Guo[1]†, Yuxiang Huang[1,2,3], Xiang Guo[1,2,3], Andreas Wagner[4,5,6]*

[1] School of Life Sciences, Westlake University, Hangzhou, China.
[2] Westlake Laboratory of Life Sciences and Biomedicine, Hangzhou, China.
[3] Institute of Biology, Westlake Institute for Advanced Study, Hangzhou, China.
[4] Department of Evolutionary Biology and Environmental Studies, University of Zurich, Zurich, Switzerland.
[5] Swiss Institute of Bioinformatics, Lausanne, Switzerland.
[6] The Santa Fe Institute, Santa Fe, USA.

*Corresponding author.
†Contributed equally.
Email: zhengjia@westlake.edu.cn; andreas.wagner@ieu.uzh.ch

This PDF file includes:

Figures S1 to S12
Tables S1 to S4
References

Supplementary figures

```
avGFP      M-SKGEELFTGVVPILVELDGDVNGHKFSVSGEGEGDATYGKLTLKFICTTGKLPVPWPT 59
GFP        MMSKGEELFTGVVPILVELDGDVNGHKFSVSGEGEGDATYGKLTLKFICTTGKLPVPWPT 60
           * **********************************************************

avGFP      LVTTFSYGVQCFSRYPDHMKQHDFFKSAMPEGYVQERTIFFKDDGNYKTRAEVKFEGDTL 119
GFP        LVTTFSYGLQCCARYPDHMKLHDFFKSAMPEGYVQERTIFFKDDGNYKTRAEVKFEGDTL 120
           ********:** :******* ***************************************

avGFP      VNRIELKGIDFKEDGNILGHKLEYNYNSHNVYIMADKQKNGIKVNFKIRHNIEDGSVQLA 179
GFP        VNRIELKGIDFKEDGNILGHKLEYNYNSHNVYIMADKQKNGIKVNFKIRHNIEDGSVQLA 180
           ************************************************************

avGFP      DHYQQNTPIGDGPVLLPDNHYLSTQSALSKDPNEKRDHMVLLEFVTAAGITHGMDELYK  238
GFP        DHYQQNTPIGDGPVLLPDNHYLSCQSALSKDPNEKRDHMVLLEFVTAAGITLGMDELYK  239
           *********************** *************************** *******
```

Supplementary Fig. 1| Sequence alignment of the *Aequorea victoria* green-fluorescent protein (avGFP) and the GFP we used, which was evolved from YFP at 37 °C in *E.coli* in a previous study [1]. Compared to avGFP, our GFP has a methionine insertion at the second residue, and six amino acid substitutions at residues 68, 71, 72, 80, 203 and 231, respectively.

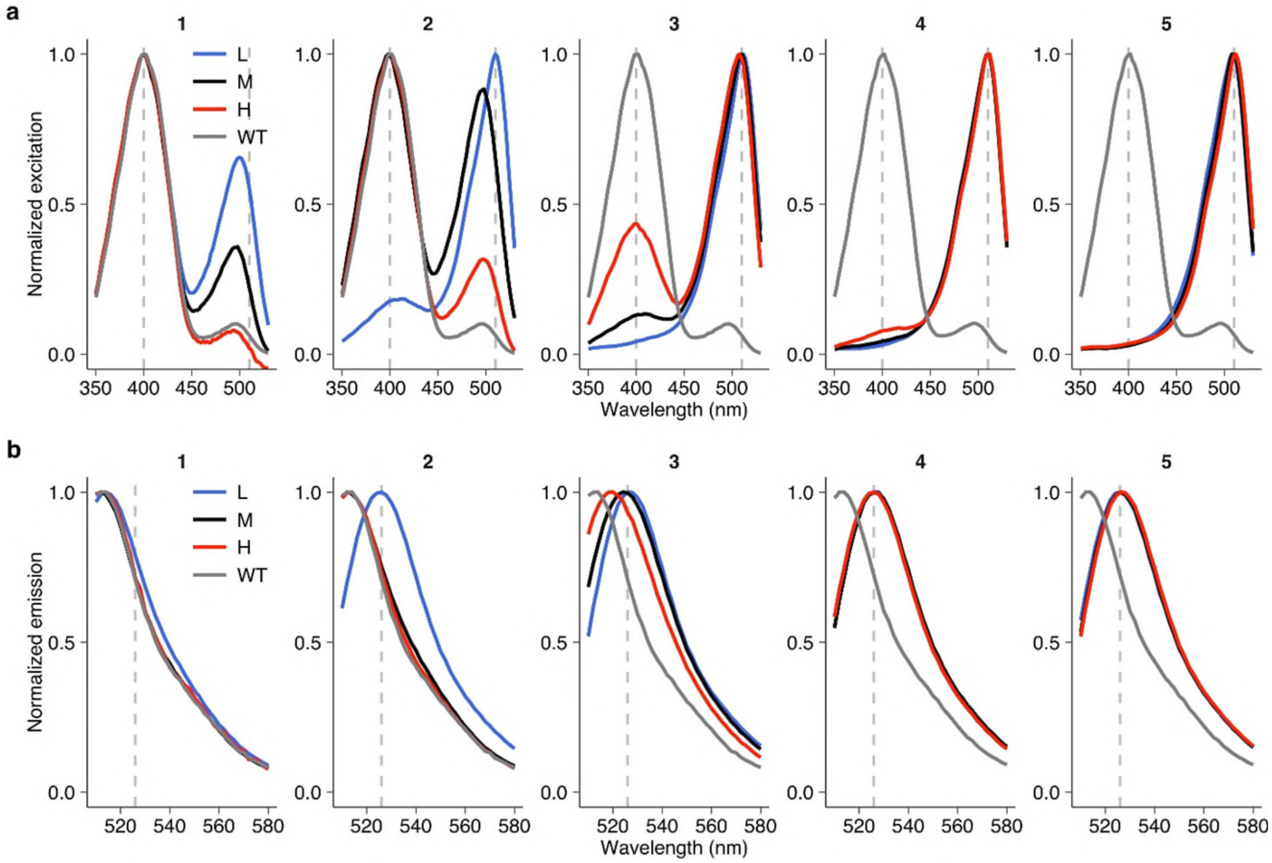

Supplementary Fig. 2| Lowered temperature promotes the evolution of new excitation and emission peaks. The letters *L*, *M* and *H* represent populations evolved at 25 °C, 37 °C and 44 °C, respectively. WT indicates ancestral GFP. Each panel shows data of normalized excitation (a) and emission (b) spectra for WT and populations *L*, *M* and *H* from generation 1 to 5, as indicated by lettering on the top. The vertical axes indicate the relative fluorescence intensity at a given emission or excitation wavelength (horizontal axis) relative to the maximal fluorescence intensity measured over the selected range of excitation or emission wavelengths. Each curved line shows the average emission spectrum of three replicate populations expressing ancestral GFP (grey), and the four replicate *L* (blue), *M* (black) or *H* (red) populations.

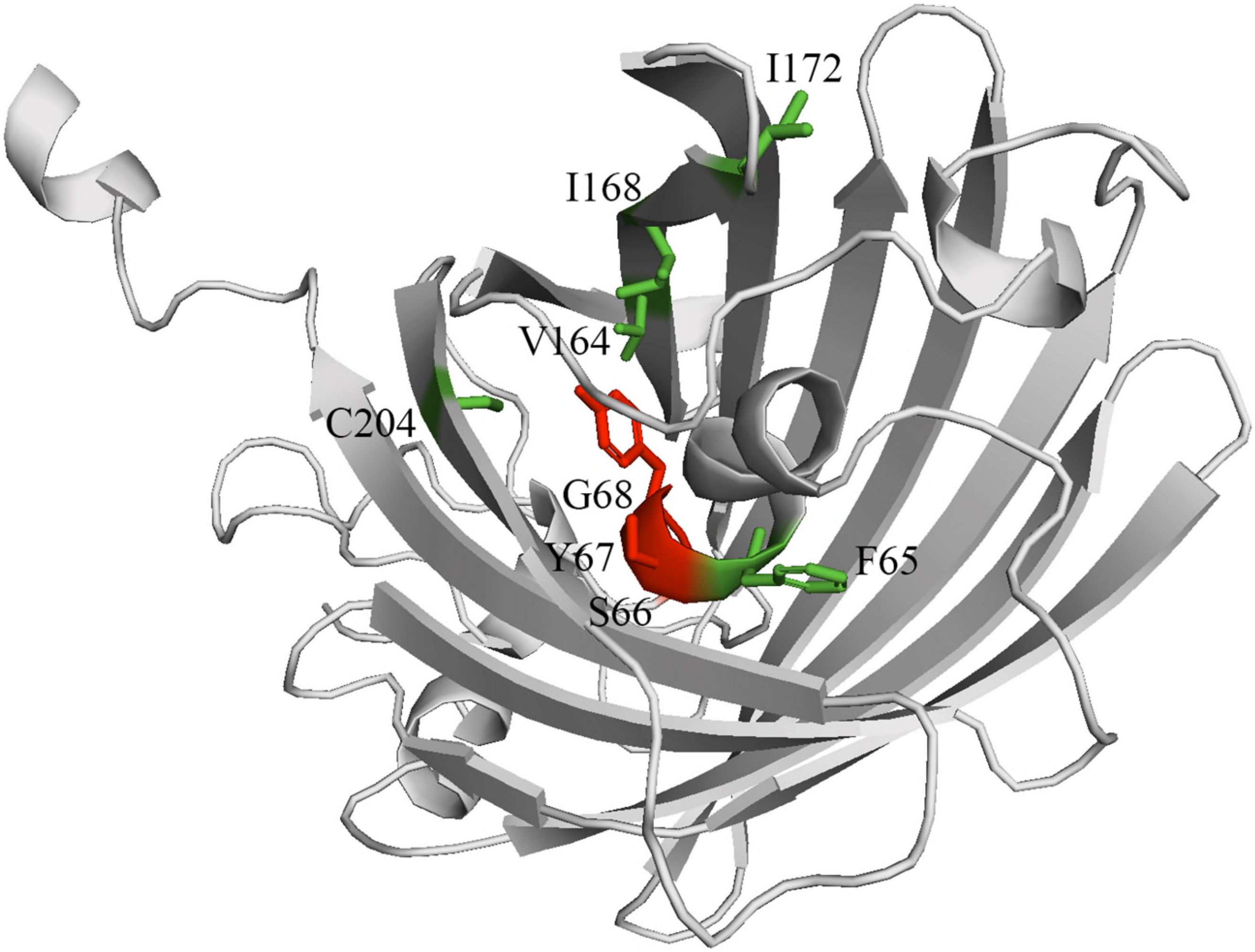

Supplementary Fig. 3 The spatial distribution of mutations that shift the excitation/emission spectra (S66G, I168V, I168T and C204Y) and that enhance folding stability (F65L, V164A and I172V) in the structure of ancestral GFP. We used ColabFold [2,3] to generate a protein model for ancestral GFP. The three residues highlighted in red (G66, Y67 and G68) can form the GFP chromophore after maturation[4].

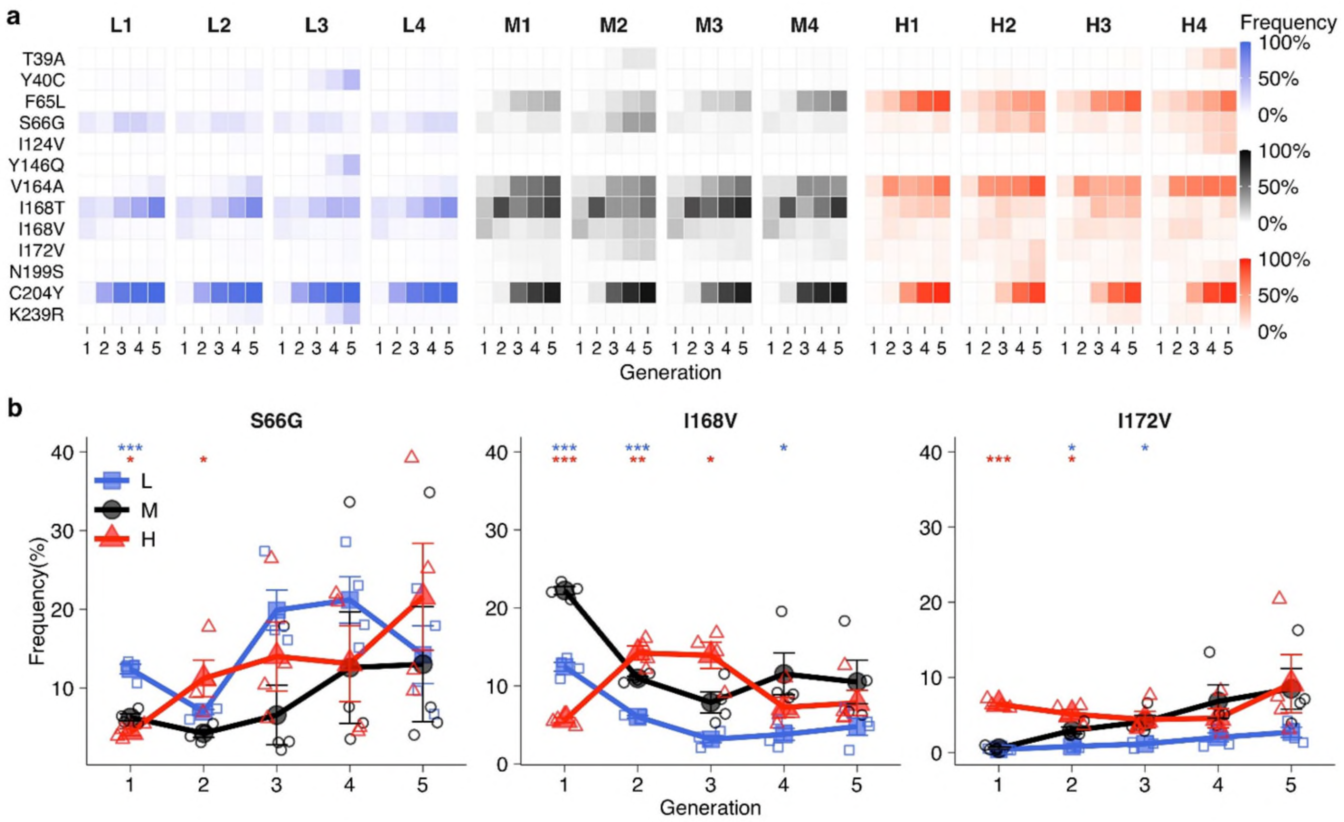

Supplementary Fig. 4| (a) Frequencies of amino acid changing mutations in evolving populations during each generation of evolution. Each of the twelve panels shows data from one replicate *L*, *M* or *H* population, as indicated by lettering on top of each panel. The horizontal axis of each panel indicates time in generations of directed evolution, and the vertical axis indicates individual mutations. Colored squares indicate mutation frequencies (see color legend). Here we only show mutations that attained a frequency exceeding 20% in at least one replicate *L*, *M* or *H* population. **(b) Frequency dynamics of high-frequency mutations S66G, I168V and I172V during evolution.** Each panel indicates frequency dynamics of specific mutations (indicated by lettering on top of each panel) during evolution. Bar height and error bars represent mean ± SEM from four replicate populations (single small colored symbols, as indicated in the color legend). **P* <0.05; ****P* <0.001 (one-way ANOVAs with Dunett's post hoc test to compare *M* (black) with *H* (red) or *L* (blue)).

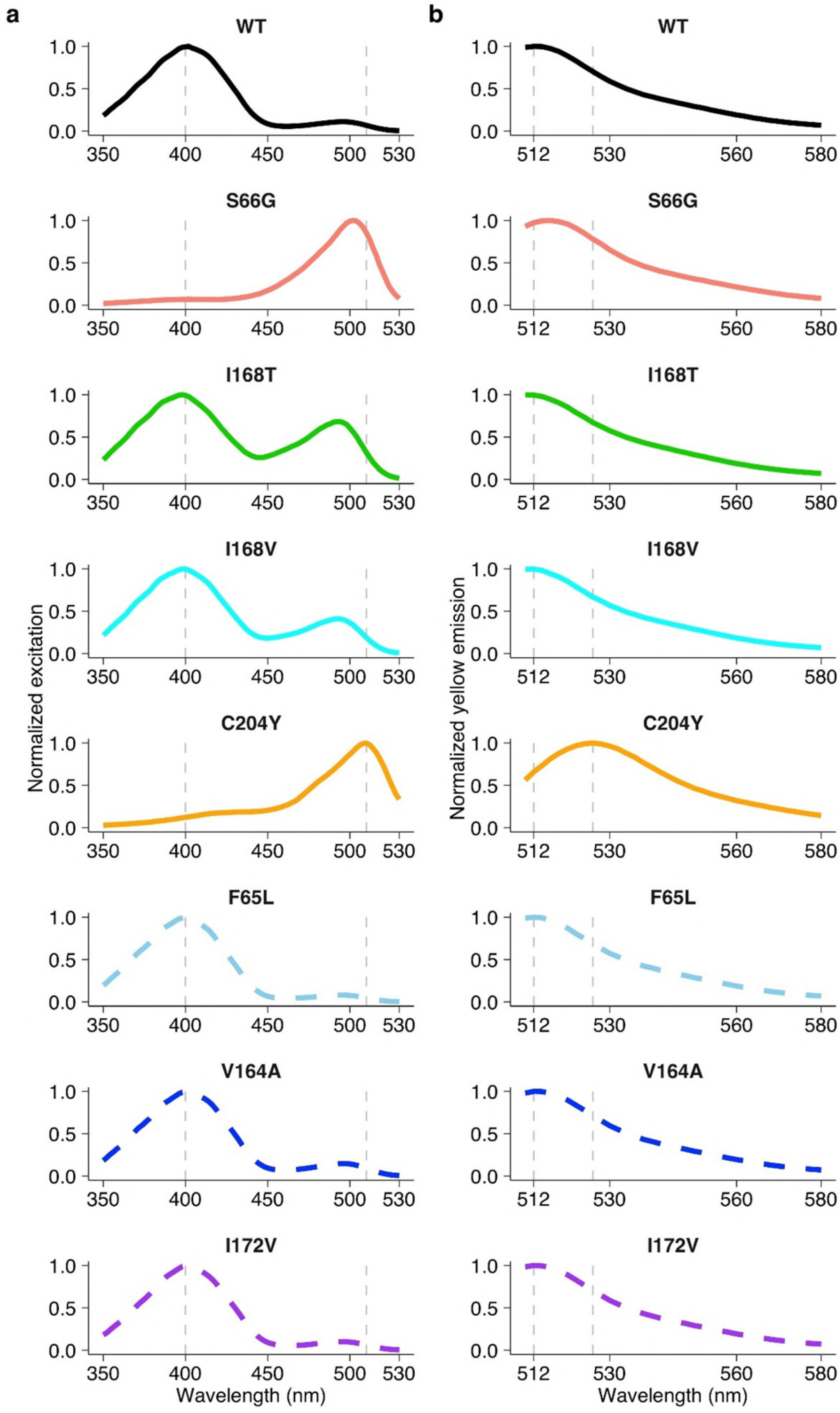
a
b
WT
S66G
I168T
I168V
C204Y
F65L
V164A
I172V
Normalized excitation
Normalized yellow emission
1.0
0.5
0.0
350
400
450
500
530
512
530
560
580
Wavelength (nm)

Supplementary Fig. 5| Excitation (a) and emission (b) spectra of high-frequency mutants. The vertical axes indicate the relative fluorescence intensity at a given emission or excitation wavelength (horizontal axes) relative to the maximal fluorescence intensity measured over the selected range of excitation or emission wavelengths. Each curved line shows the average emission spectrum of ancestral GFP (WT) or of each mutant based on three biological replicate measurements.

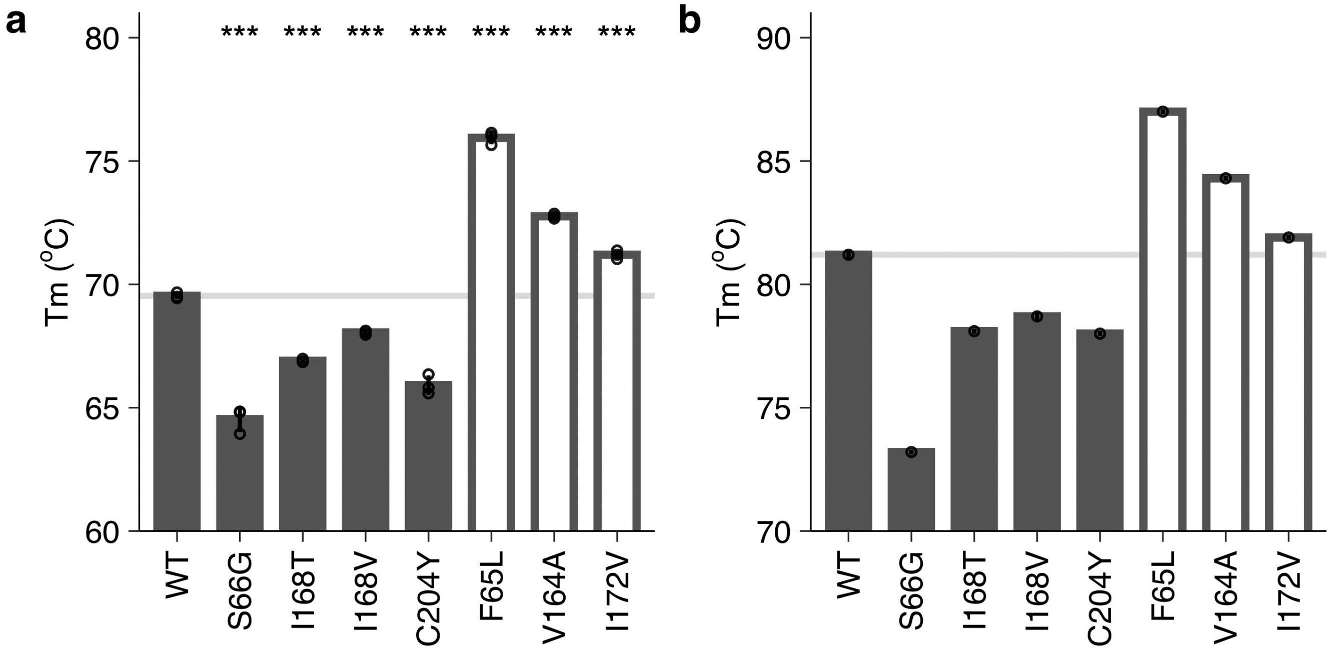

Supplementary Fig. 6| Melting temperatures (*Tm*) of ancestral GFP (WT) and high-frequency mutants as estimated from (a) cell lysates and (b) purified fluorescent proteins (see Methods). We performed one-way ANOVAs with Dunnett's post-hoc test to ask whether each mutant's *Tm* was significantly different from that of ancestral GFP (WT) in panel a. Bar height and error bars represent mean ± SD based on three biological replicate measurements (open circles) in panel a. ***$P < 0.001$.

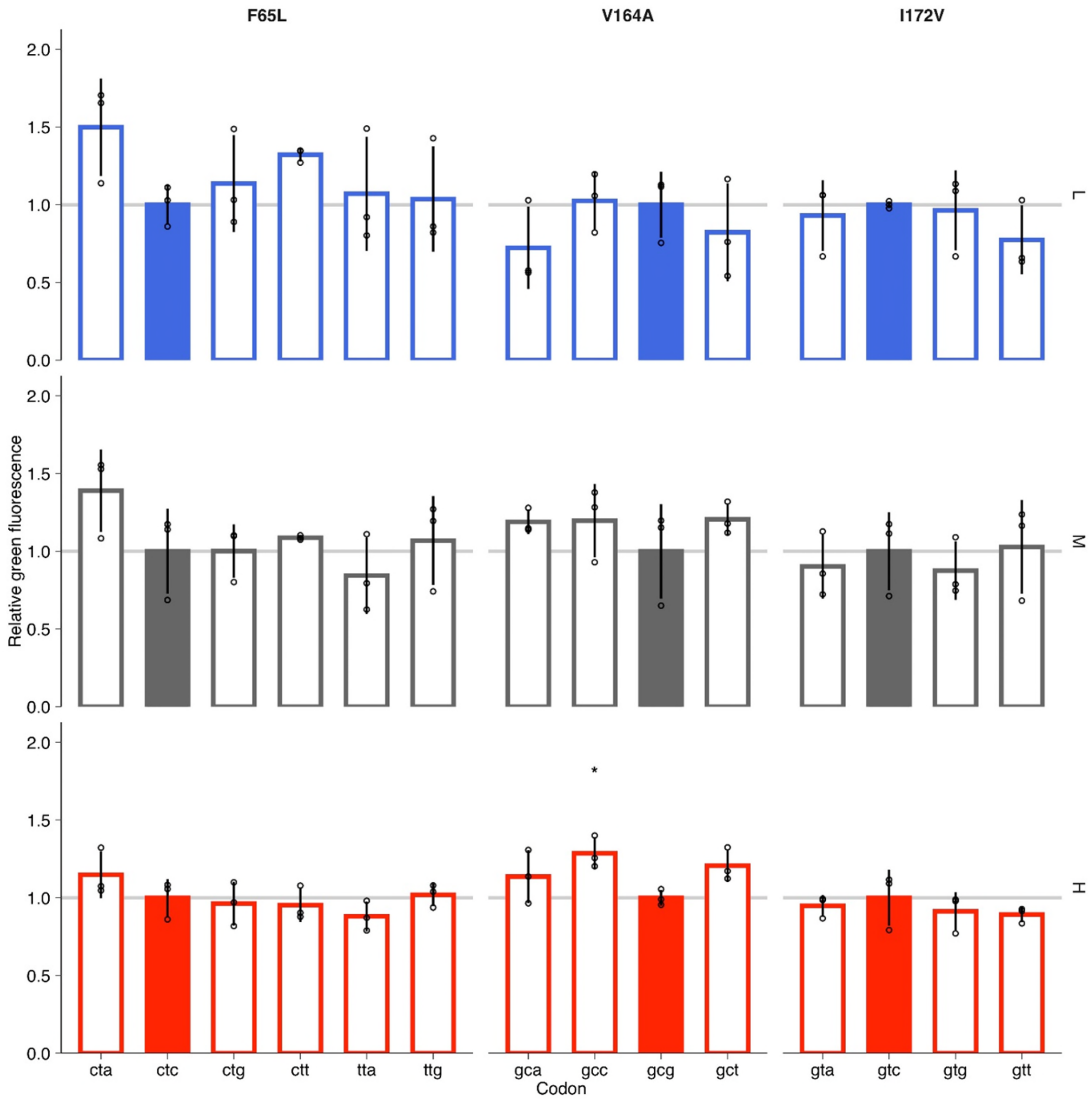

Supplementary Fig. 7| Synonymous mutations in each of the three stabilizing mutants (as labeled on the top of each panel) do not substantially change green fluorescence at low, medium, and high temperature. In each panel, the vertical axis indicates green fluorescence of each synonymous mutation (open bars), as indicated by the corresponding synonymous codon (horizontal axis), relative to the original genotype that occurred during experimental evolution (filled bars, horizontal grey line). We performed one-way ANOVAs with Dunnett's post hoc test to test the null hypothesis that the relative fluorescence intensity of each synonymous mutation is identical to the original fluorescence at low (*L*,

blue), medium (*M*, grey) and high temperatures (*H*, red). Bar height and error bars represent mean ± SD based on three biological replicate measurements (open circles). *$P < 0.05$.

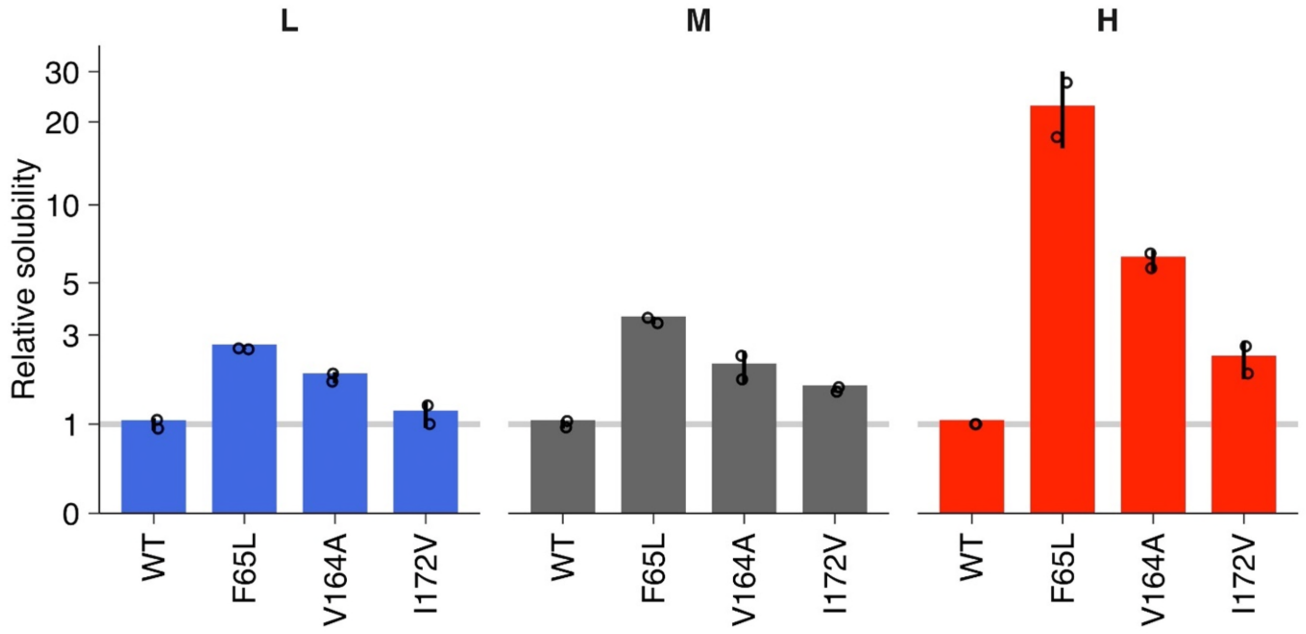

Supplementary Fig. 8| Relative solubility of stabilizing mutants, as estimated by cell-free expression at low (*L*), medium (*M*) and high (*H*) temperatures (see methods). The vertical axis indicates the amount of soluble protein for each mutant (horizontal axis) relative to ancestral GFP (WT, horizontal grey line). Bar height and error bars represent mean ± SD based on two biological replicate measurements (small open circles).

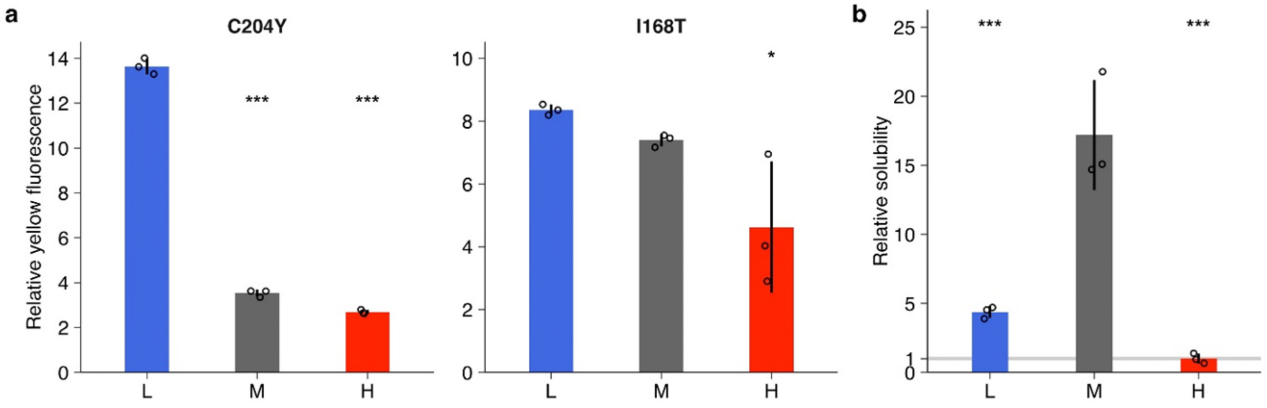

Supplementary Fig. 9| (a) Fitness effects of neofunctionalizing mutations C204Y and I168T at different temperatures. The vertical axes indicate the fluorescence intensity of mutations C204Y **(**left**)** and I168T **(**right**)** relative to ancestral GFP at the corresponding temperature (horizontal axes). **(b) Effects of temperature on soluble expression of ancestral GFP.** We used an ELISA to quantify the amount of soluble fluorescent protein of ancestral GFP, and normalized the resulting solubility values at different expression temperatures (horizontal axis) by dividing it by the cell density (OD600, see 'Methods') to account for variation in cell numbers among cultures. We then divided the resulting normalized solubility for each temperature by that at the high temperature to calculate the solubility at each temperature relative to that at high temperature (vertical axis). Bar height and error bars represent mean ± SD based on three biological replicate measurements (single small symbols). We performed one-way ANOVA with Dunnett's post hoc test to test the null hypothesis that the relative fluorescence intensity of C204Y or I168T at high temperature (*H*, red) or low temperature (*L*, blue) is identical to that at medium temperature (*M*, grey) in panel a. We used the same strategy to compare the significant difference in solubility between *M* (grey) and *H* (red) or *L* (blue) in panel b. * and *** indicate $P < 0.05$ and 0.001, respectively.

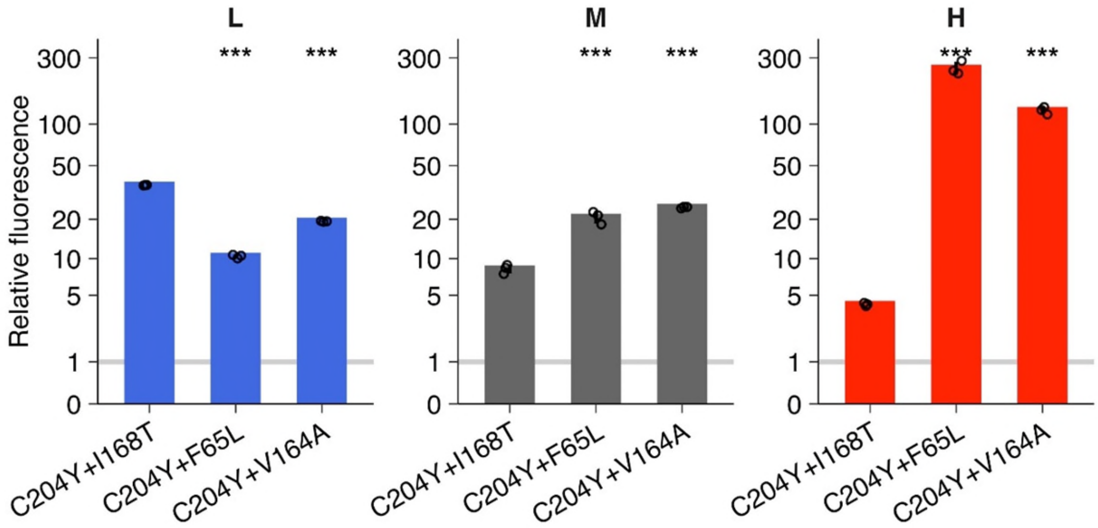

Supplementary Fig. 10 Temperature can modify fitness effects of high-frequency double mutants. The vertical axes indicate the yellow fluorescence intensity of each double mutant (horizontal axes) relative to ancestral GFP at the corresponding temperature. Bar height and error bars represent mean ± SD based on three biological replicate measurements (single small symbols). We performed one-way ANOVA with Dunnett's post hoc test to ask whether the yellow fluorescence for the double mutant C204Y+I168T was significantly different from that for C204Y+F65L or C204Y+V164A at the corresponding temperature. ****P* < 0.001.

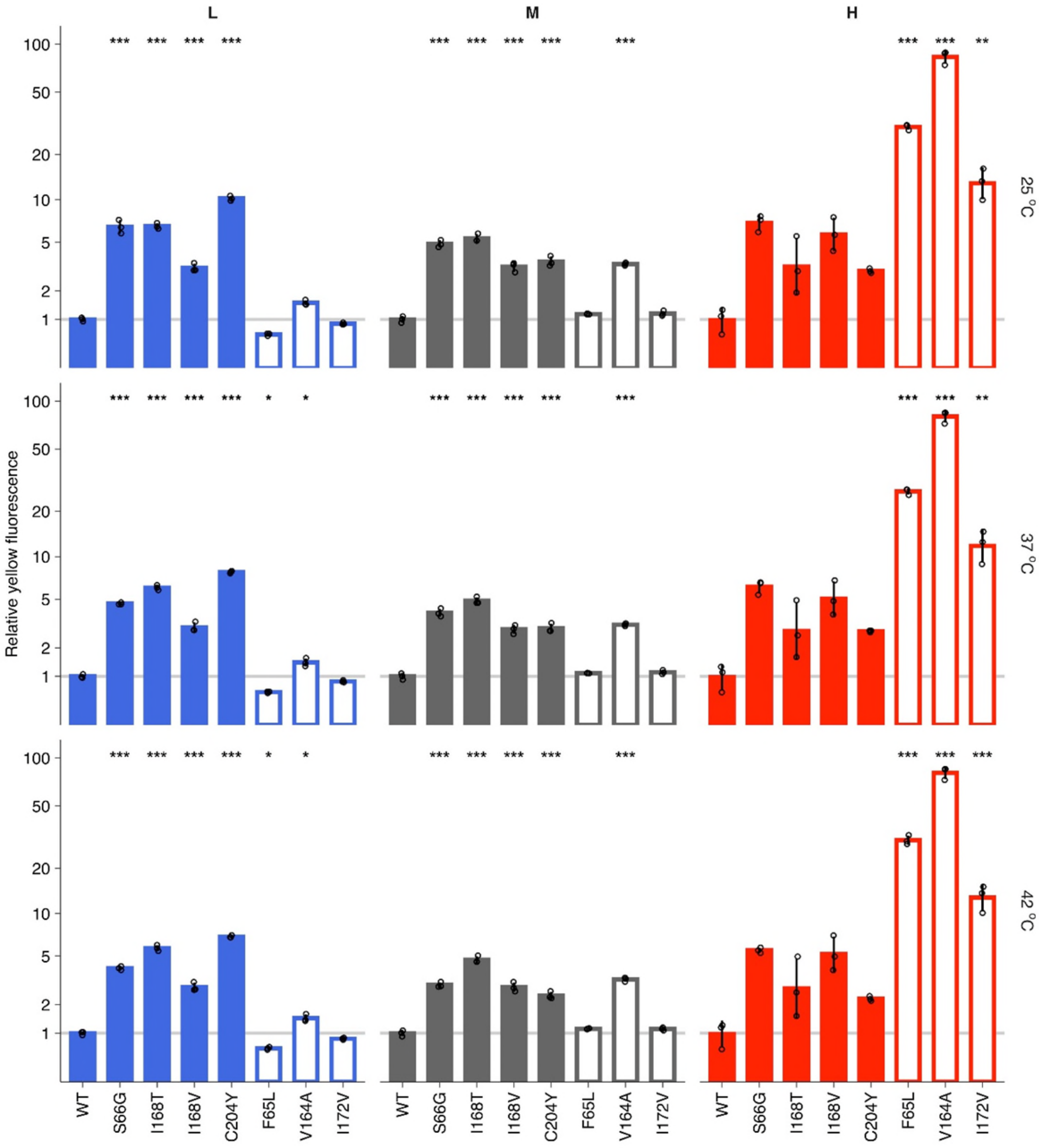

Supplementary Fig. 11| Yellow fluorescence of GFP variants relative to ancestral GFP (WT) measured at different temperatures. Note the logarithmic vertical scales. We expressed each mutant (horizontal axes) at low (*L; blue*), medium (*M; grey*) and high (*H; red*) temperatures, and then measured yellow fluorescence intensities at 25 °C (top), 37 °C (middle) and 42 °C (bottom) by using a Tecan plate reader. Bar height and error bars represent mean ± SD based on three biological replicate measurements. We performed one-way ANOVAs with Dunnett's post hoc test to test the null hypothesis that the

relative fluorescence intensity of a given mutant is identical to that of ancestral GFP (WT) at the corresponding temperatures. *, ** or *** indicate $P < 0.05$, 0.01 or 0.001, respectively.

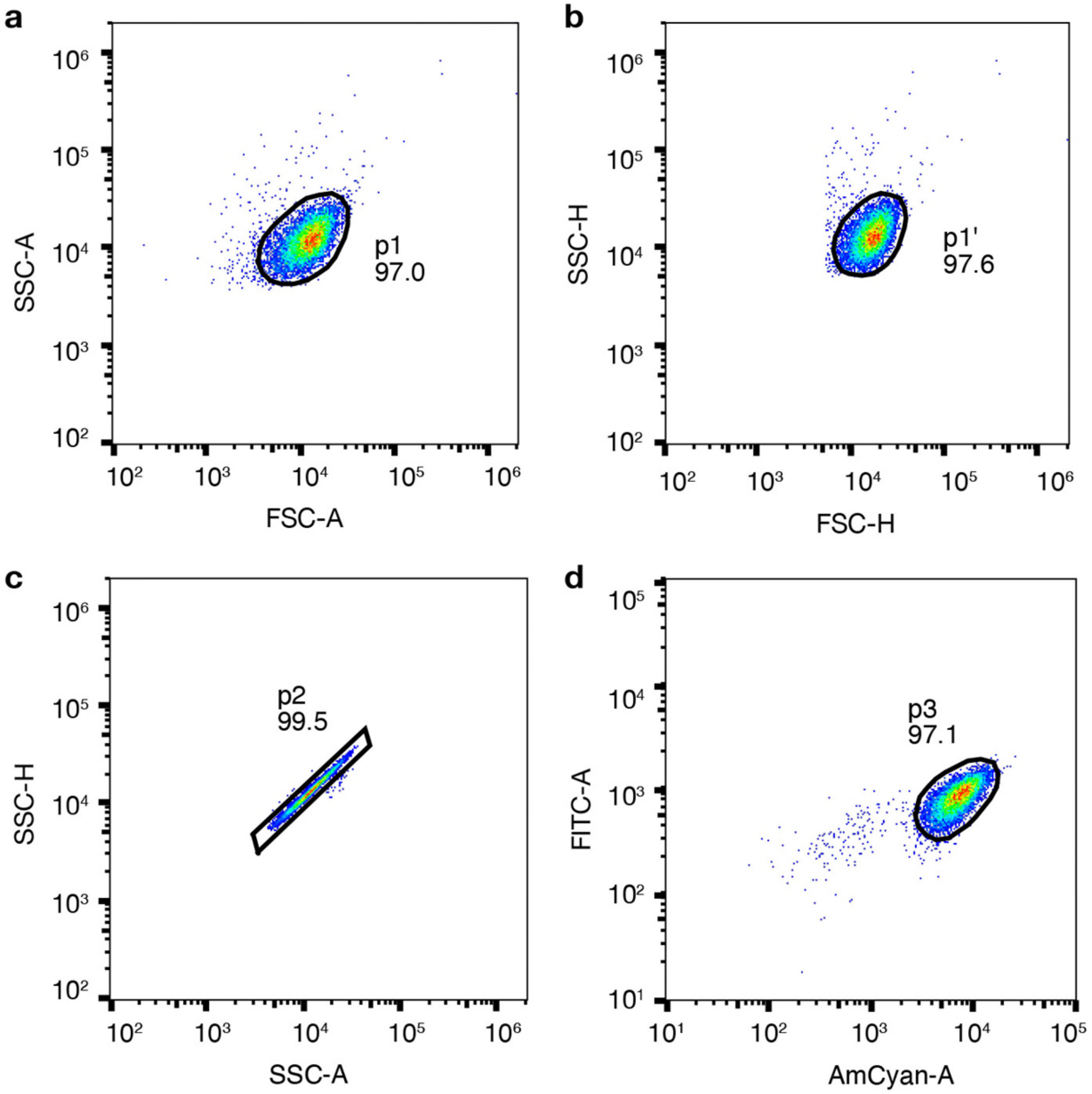

Supplementary Fig. 12| Gating strategies for flow cytometry data analysis. We selected a homogenous cell population p1 (panel a) from the FSC-H (forward scatter height) versus SSC-H (side scatter height) density plot or or p1' (panel b) from the FSC-A (forward scatter area) versus SSC-A (side scatter area) density plot. We then excluded doublets by using the SSC-A (side scatter area) versus SSC-H (side scatter height) density plot to select a singlet population p2 (panel c). We next used the FITC-Area versus AmCyan-Area density plot to select the dominant cell population p3 (panel d) for calculating the mean fluorescence intensity of each biological replicate for engineered variants. We

directly used the resulting filtered data p2 for estimating the mean fluorescence intensity of each evolving replicate population.

Supplementary tables

Supplementary Table 1 Mean number of DNA mutations per GFP molecule for each evolving population during each generation of evolution

Population	Number of DNA mutations per read in each generation				
	1	2	3	4	5
L1	1.65	2.59	3.77	4.99	6.38
L2	1.63	2.52	3.59	5.08	6.40
L3	1.69	2.60	4.04	5.70	7.59
L4	1.70	2.53	3.58	4.86	6.05
M1	1.53	2.73	3.98	5.65	6.77
M2	1.56	2.75	4.60	5.86	6.65
M3	1.52	2.74	4.15	5.55	6.35
M4	1.55	2.71	3.70	5.24	6.51
H1	1.64	2.93	4.01	4.86	6.20
H2	1.62	3.04	4.37	5.29	6.62
H3	1.62	2.94	3.99	4.88	6.33
H4	1.64	2.98	4.01	5.04	6.45
Control[a]	1.98	1.28	1.24	1.45	1.37
GFP (ancestor)[b]			0.03		
			0.01		
			0.02		

[a] mutation rates, as estimated by SMRT-sequencing the mutation libraries using ancestral GFP as a template. Specifically, we incubated the mutation libraries after transformation and recovery at 37 °C with shaking at 220 rpm for 12~14 h and isolated plasmids for SMRT-sequencing.

[b] to estimate errors that occurring during sequencing library preparation and SMRT sequencing, we sequenced ancestral GFP by following the same process as for our evolving populations (see ‘Methods’).

Supplementary Table 2 Mean number of amino-acid changing mutations per GFP molecule for each evolving population during each generation of evolution

Population	Number of amino acid mutations per read in each generation				
	1	2	3	4	5
L1	1.42	2.14	3.07	3.91	4.57
L2	1.40	2.10	2.93	4.04	4.92
L3	1.45	2.14	3.23	4.31	5.31
L4	1.47	2.10	2.97	3.84	4.57
M1	1.30	2.28	3.40	4.81	5.63
M2	1.31	2.31	3.87	4.92	5.42
M3	1.28	2.26	3.42	4.50	5.03
M4	1.33	2.23	3.05	4.22	5.13
H1	1.39	2.56	3.32	4.10	5.20
H2	1.39	2.59	3.78	4.68	5.87
H3	1.33	2.45	3.43	4.16	5.39
H4	1.39	2.60	3.52	4.41	5.62
Control [a]	1.75	1.09	1.07	1.23	1.16
GFP (ancestor) [b]			0.02<br>0.01<br>0.02		

[a] mutation rates, as estimated by SMRT-sequencing the mutation libraries using ancestral GFP as a template. Specifically, we incubated the mutation libraries after transformation and recovery at 37 °C with shaking at 220 rpm for 12~14 h and isolated plasmids for SMRT-sequencing.

[b] to estimate errors that occurring during sequencing library preparation and SMRT sequencing, we sequenced ancestral GFP by following the same process as for our evolving populations (see 'Methods').

Supplementary Table 3 Number of SMRT sequencing reads for each evolving populations during each generation of evolution

Population	Number of reads in each generation				
	1	2	3	4	5
L1	2788	2367	2278	2673	2370
L2	3338	2287	2922	2255	2040
L3	2603	2099	1967	1878	1859
L4	1199	1155	1504	1085	1076
M1	2500	3879	1591	2224	1666
M2	2439	4278	1725	2393	1707
M3	2773	5303	2940	2308	1758
M4	2925	2972	3104	1725	1515
H1	2660	2540	3301	2248	1784
H2	2287	2796	2696	2149	1733
H3	2992	3197	2418	2066	1694
H4	3436	2705	2624	2856	1783
Control[a]	1578	2533	2473	2563	1306
GFP (ancestor)[b]	2017<br>2217<br>1449				

[a] mutation rates, as estimated by SMRT-sequencing the mutation libraries using ancestral GFP as a template. Specifically, we incubated the mutation libraries after transformation and recovery at 37 °C with shaking at 220 rpm for 12~14 h and isolated plasmids for SMRT-sequencing.

[b] to estimate errors that occurred during the preparation of sequencing libraries and SMRT sequencing, we sequenced ancestral GFP by following the same process as for our evolving populations (see 'Methods').

Supplementary Table 4 Primers for barcoding PCRs [a]

Primers		Sequence
Forward primers	1F	CAGCAGATCATGTCGAgcagtcgaacatgtagctgactcaggtcac
	2F	TACGATCGTAGCTGCTgcagtcgaacatgtagctgactcaggtcac
	3F	GTACACGCTGTGACTAgcagtcgaacatgtagctgactcaggtcac
	4F	ACAGTGCGCTGTCTATgcagtcgaacatgtagctgactcaggtcac
	5F	TATCAGCACGACATGCgcagtcgaacatgtagctgactcaggtcac
	6F	ACTGCGAGATACACACgcagtcgaacatgtagctgactcaggtcac
	7F	CATACATCGCGCAGTAgcagtcgaacatgtagctgactcaggtcac
	8F	CACATATCAGAGTGCGgcagtcgaacatgtagctgactcaggtcac
	9F	GATAGCTGCTAGCTGAgcagtcgaacatgtagctgactcaggtcac
	10F	TCACTGTGTGTGTCTGgcagtcgaacatgtagctgactcaggtcac
	11F	GATCTGTCGTGAGCGTgcagtcgaacatgtagctgactcaggtcac
	12F	TATCTGAGCGCGAGCAgcagtcgaacatgtagctgactcaggtcac
	13F	CTCACGTACGTCACACgcagtcgaacatgtagctgactcaggtcac
	14F	CGTGTCGCGCATATCTgcagtcgaacatgtagctgactcaggtcac
	15F	TGTGCACGACAGCAGTgcagtcgaacatgtagctgactcaggtcac
	16F	ACGTCAGCACTGCTCTgcagtcgaacatgtagctgactcaggtcac
	17F	GACTGCACATGCACGAgcagtcgaacatgtagctgactcaggtcac
	18F	CTACGAGACAGATCGCgcagtcgaacatgtagctgactcaggtcac
Reverse primers	1R	CGAGTAGACAGTGACTtggatcacttgtgcaagcatcacatcgtag
	2R	AGTGCACGAGCATATGtggatcacttgtgcaagcatcacatcgtag
	3R	CGTGTGATGTCTACAGtggatcacttgtgcaagcatcacatcgtag
	4R	ACACGCGATCTAGTGTtggatcacttgtgcaagcatcacatcgtag
	5R	AGCGCAGTGTATAGTGtggatcacttgtgcaagcatcacatcgtag
	6R	GATGAGAGAGCTCTCTtggatcacttgtgcaagcatcacatcgtag
	7R	TATGCAGTCTGTCGTCtggatcacttgtgcaagcatcacatcgtag
	8R	TCACGCTCTGTCTACTtggatcacttgtgcaagcatcacatcgtag
	9R	GCGATCTAGCTATGTCtggatcacttgtgcaagcatcacatcgtag
	10R	GCTATACGCTCGATACtggatcacttgtgcaagcatcacatcgtag
	11R	GTGACTGCGTGTCTAGtggatcacttgtgcaagcatcacatcgtag
	12R	CGAGTGCTAGACGATGtggatcacttgtgcaagcatcacatcgtag
	13R	ACGCACTATGACGTCGtggatcacttgtgcaagcatcacatcgtag
	14R	CTGTCAGAGTAGCTCGtggatcacttgtgcaagcatcacatcgtag
	15R	TCGCGATAGTCTCGCAtggatcacttgtgcaagcatcacatcgtag

16R	GATGCTCGAGTCGATCtggatcacttgtgcaagcatcacatcgtag
17R	GTGAGCGCAGTGAGTAtggatcacttgtgcaagcatcacatcgtag
18R	ACAGATGTCTGTGCGCtggatcacttgtgcaagcatcacatcgtag
tsmrtF	gcagtcgaacatgtagctgactcaggtcacCTTTAAGAAGGAGATATACCTCGAG
tsmrtR	tggatcacttgtgcaagcatcacatcgtagAATCTTCTCTCATCCGCC

[a] Sequences in upper case indicate barcoding regions for all 18 forward and 18 reverse barcoding primers. For primers tsmrtF/tsmrtR, sequences in upper case indicate DNA located in flanking regions of the *gfp* gene.

References

1. Zheng, J., Payne, J. L. & Wagner, A. Cryptic genetic variation accelerates adaptive evolution by opening access to diverse adaptive peaks. *Science.* **365**, 347–353 (2019).

2. Mirdita, M. *et al.* ColabFold: making protein folding accessible to all. *Nat. Methods 2022 196* **19**, 679–682 (2022).

3. Jumper, J. *et al.* Highly accurate protein structure prediction with AlphaFold. *Nat. 2021 5967873* **596**, 583–589 (2021).

4. De Meulenaere, E. *et al.* Improving the second-order nonlinear optical response of fluorescent proteins: The symmetry argument. *J. Am. Chem. Soc.* **135**, 4061–4069 (2013).
